# Atypical Lyme Disease Rash: A Case Report

**DOI:** 10.7759/cureus.54779

**Published:** 2024-02-23

**Authors:** Selena Khanna, Lynne J Goebel

**Affiliations:** 1 Internal Medicine, Joan C. Edwards School of Medicine, Marshall University, Huntington, USA

**Keywords:** atypical presentation, homogeneous purple rash, bullseye rash, borrelia burgdorferi, erythema migrans, lyme disease

## Abstract

Lyme disease (LD), caused in the United States primarily by *Borrelia burgdorferi *sensu lato, is a tick-borne illness characterized by a spectrum of clinical manifestations depending on the stage of illness. Most clinicians are familiar with the classic bullseye appearance of the erythema migrans (EM) rash that occurs in the early stage of the disease. However, many providers may not be aware of alternate appearances for the rash. This paper reports the case of a 69-year-old female with LD, exhibiting an atypical rash with purplish discoloration that was devoid of an outer ring or central clearing. In geographic areas with a high incidence of LD, it is especially important for clinicians to recognize alternative LD presentations. Healthcare providers should maintain a high index of suspicion of LD in patients with tick bites, even without typical EM, to ensure early diagnosis and treatment. Education on diverse LD presentations is crucial for improving public health outcomes.

## Introduction

According to the CDC, surveillance data reveals that 476,000 people test positive for Lyme disease (LD) each year in the United States [1]. Most clinicians recognize LD by the typical associated bullseye appearance of the erythema migrans (EM) rash. However, a diagnostic challenge occurs when an alternate appearance for the rash is present. Limited recognition of an atypical LD rash underscores the need for increased awareness among healthcare providers. In one study of 69 people who were part of a Lyme Disease Biobank database and presented to a medical center in Wisconsin, a high LD incidence area, over half of the EM lesions were homogeneous in color and only six percent exhibited a bullseye [2]. Knowledge of atypical rash appearances is crucial for healthcare practitioners, working toward improving the management of LD with treatment in the early stage as this prevents long-term morbidity in the later stages of the disease. We present the case of a 69-year-old native West Virginia female with LD who exhibited a rash without the classic outer ring or inner clearing to emphasize the importance of recognizing the diversity in EM presentations, given that atypical forms may delay accurate diagnosis and prompt treatment. Education is vital in states such as West Virginia where LD has the second-highest incidence rate in the United States [1]. This case was presented on November 10, 2023, at the West Virginia American College of Physicians annual meeting in Stonewall, West Virginia.

## Case presentation

**Figure 1:**
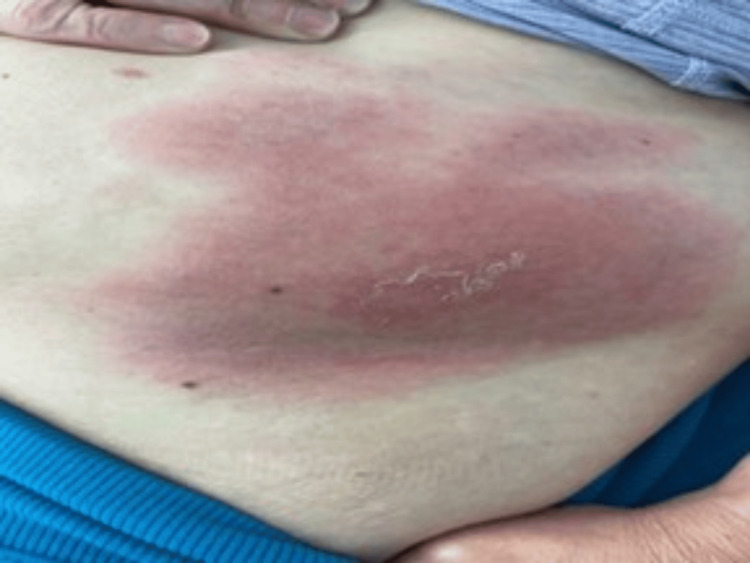
Purplish lesion on the left abdominal wall

A 69-year-old West Virginian female, with a history of degenerative joint disease, asthma, breast cancer, and hypothyroidism, sought medical attention because of an itchy rash on her abdomen, which developed six days following a tick bite. On physical exam, her vital signs were normal. Purplish, papular, oval lesions up to 12 centimeters in size were noted on the left lower abdominal wall, spreading from a central area of scaling assumed to be the location of the tick bite (Figure 1).

**Figure 2:**
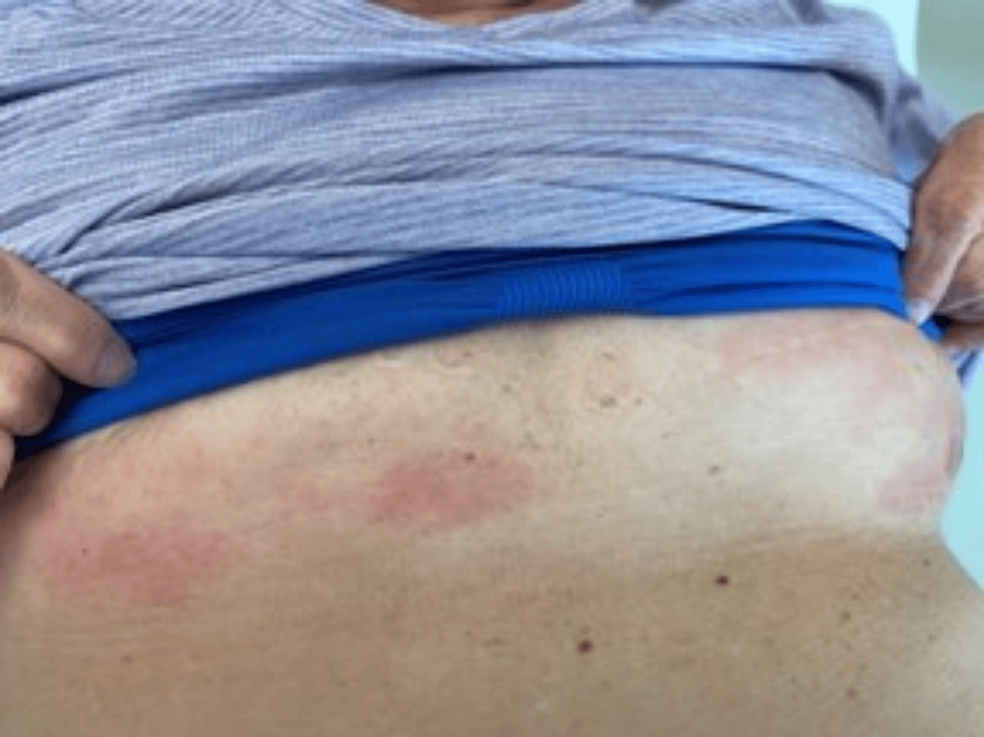
Purplish lesion seen under the breasts

**Figure 3:**
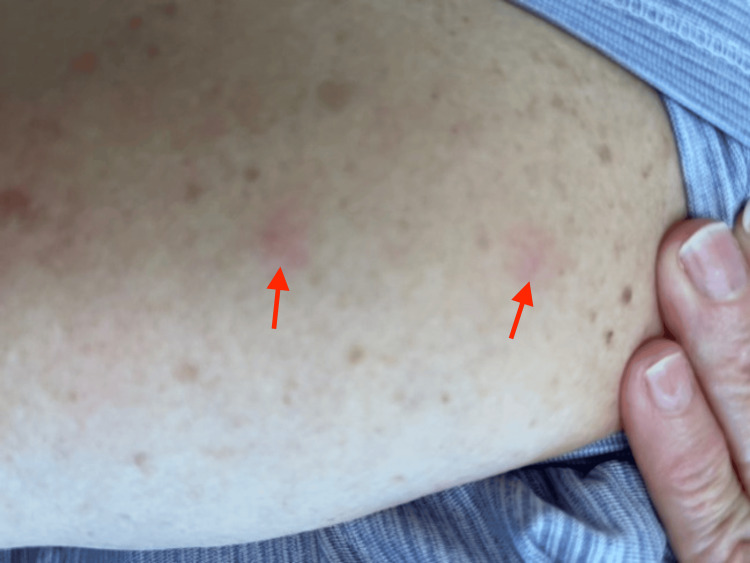
Erythematous papules (red arrows) seen along the right arm

Additionally, the patient had erythematous papular lesions underneath the breasts bilaterally (Figure 2) and a couple of smaller erythematous papules along the right upper arm approximately 1.0 cm in diameter (Figure 3).

**Figure 4:**
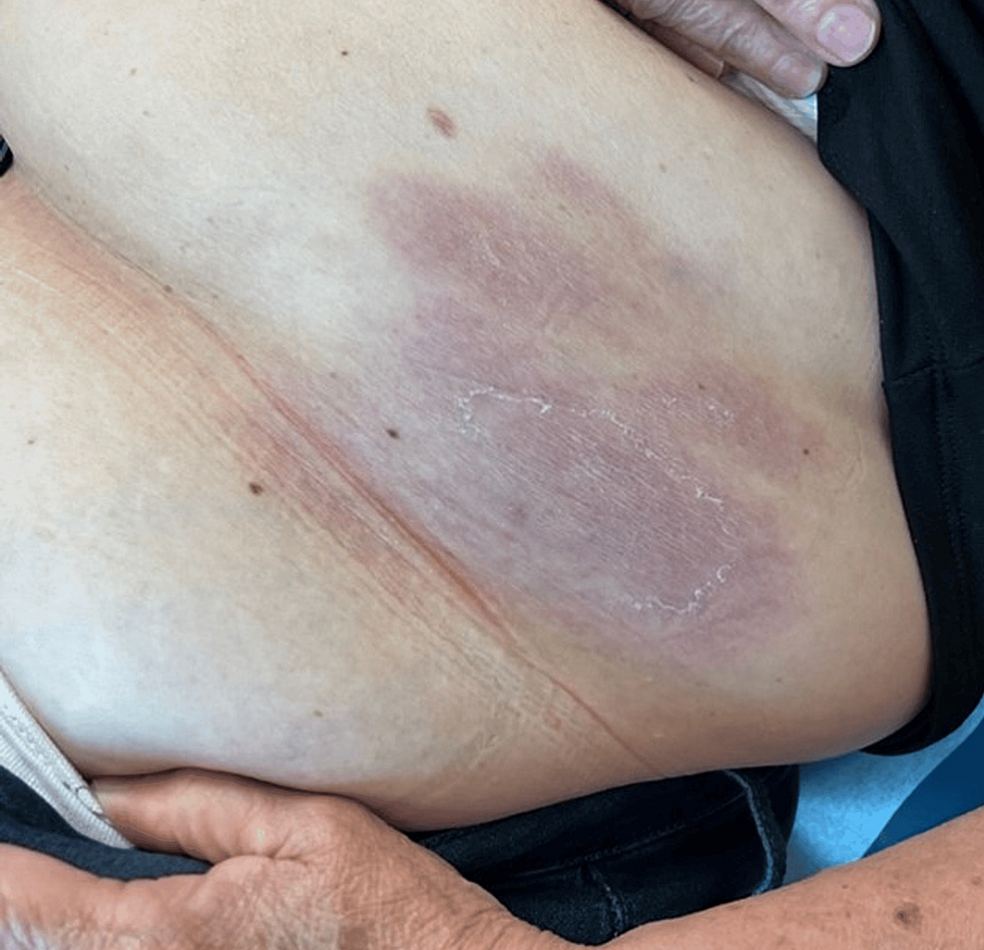
Fading of the erythematous lesion on the left abdominal wall

**Figure 5:**
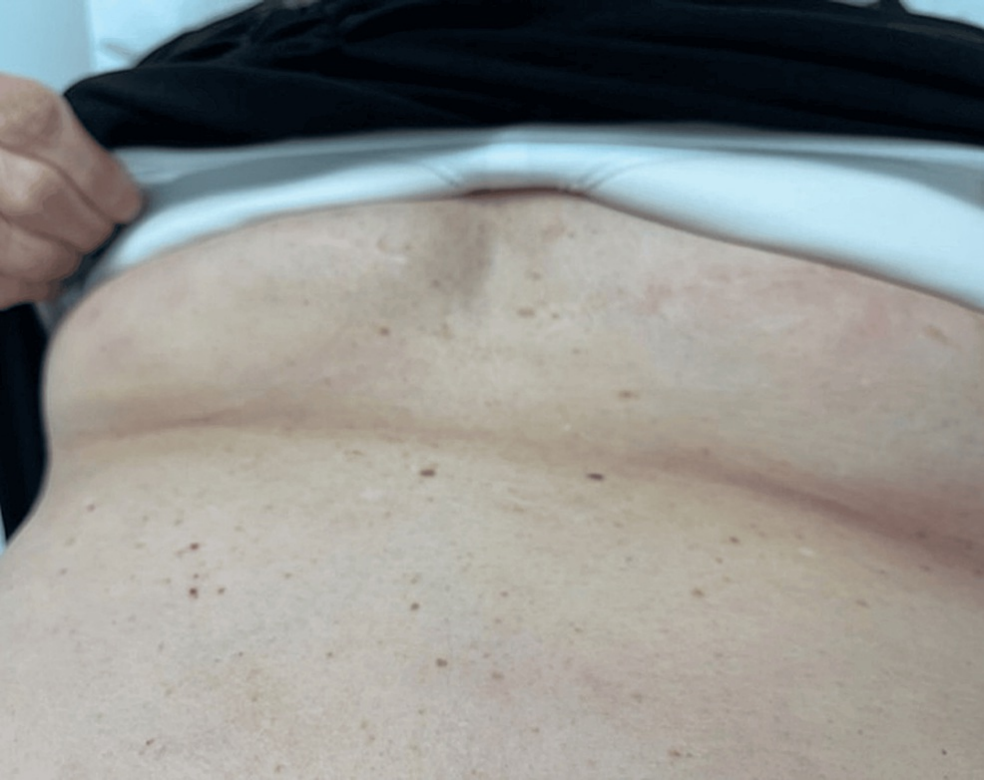
Fading of the erythematous lesion under the breasts

Serologic testing confirmed Lyme IgM by ELISA, but the confirmatory Western blot test was negative, showing only one band of IgG P58 antibodies. She received doxycycline 100 mg twice daily for 14 days empirically before test results, and the patient returned a week later with significant improvement of the rash and visible fading of erythema in the abdominal region with no additional symptoms (Figures 4, 5).

## Discussion

This patient had LD, the most prevalent vector-borne disease in the United States [1]. With a rate of 100.14 new cases per 100,000 residents in 2021, West Virginia has one of the highest LD incidence rates in the nation [1]. Lyme borreliosis classically begins with skin lesions enlarging at the location of the tick bite. The disease follows three main stages: early localized, early disseminated, and chronic disseminated, with more than half of untreated patients advancing to disseminated disease [3]. *Borrelia burgdorferi *spirochetes, the bacteria causing LD, can persist in organs for months to years after the initial infection, leading to arthritis, carditis, and meningitis, and, in at least one case, was reported to persist after antibiotic treatment [4]. There may be a chronic form of illness occurring after antibiotic treatment causing chronic fatigue [3].

Diagnosis of early LD relies on the clinical recognition of dermatologic and physical manifestations of EM early in the disease, and laboratory testing is not necessary before beginning treatment [3]. Serologic testing for LD is performed via an initial ELISA test positive for IgM, followed by a confirmatory Western blot test that is usually positive if more than five bands are present [5]. However, the Western blot test has limitations that make the diagnosis of LD in its early stages challenging as it may yield a negative result early in the disease. ELISA may result in false positives given the cross-reactivity that occurs when antibodies react to different bacteria that may share a common protein [5]. Our patient only had one band positive on Western blot testing, which is not considered a positive result. Our testing was likely too early to show a positive result.

As EM can present uncharacteristically, its recognition by clinicians can be challenging. In West Virginia, 2021 data revealed that only 49% of cases had the EM rash [6]. While LD is best known to be associated with a bullseye rash, understanding the atypical presentations of this disease is imperative to ensure early diagnosis and antibiotic management, which prevents the chronic disseminated stage of the disease. In a study conducted in 2012 designed to understand the recognition of various appearances of LD by users of the Lyme Disease Research Foundation website, only 20.5% of participants could correctly identify a non-bullseye EM rash, while 72.7% were able to correctly identify the bullseye rash [7]. Study results showed that 25.7% of the participants incorrectly identified the uniformly red EM, 43.6% incorrectly identified the disseminated EM, and 33.0% incorrectly identified the blue-purple EM presentation that was similar to our patient’s rash [7].

A purple rash from a tick bite is not a typical presentation. In Schotthoefer et al.’s series, only six percent of the EM rashes were purple [2]. In rare cases, certain tick-borne diseases or complications from tick bites may lead to skin changes that could appear purple or blue. Some of the most common conditions that can cause this aside from LD include Rocky Mountain spotted fever (RMSF) and disseminated intravascular coagulation (DIC) [8]. RMSF is primarily associated with a maculopapular rash with red spots and petechiae caused by bleeding under the skin. This can cause a purple appearance but not usually a solid purple rash. Typically, the RMSF rash starts in the wrists and spreads inward toward the trunk and, later, you can see lesions on the palms and soles that are characteristic of the disease [8]. The lesions are typically small, less than 5 mm, and progress to purpuric lesions. It is important to distinguish LD from RMSF as it is also seen in West Virginia, although RMSF is not nearly as common with only 12 cases reported in 2021 compared to 1,788 cases of LD that same year [7]. Southern tick-associated rash illness (STARI) is associated with an EM rash that could be confused with LD, but it does not cause chronic illness and is not seen in West Virginia [6,8]. Tick bites can sometimes trigger systemic reactions that can cause coagulation with the formation of small blood clots throughout the body leading to skin changes and purple discoloration such as DIC [8]. Scurvy, vasculitis, and idiopathic thrombocytopenia purpura can also cause a purple rash and would be included in the differential diagnosis [9].

In geographic areas where LD is highly prevalent, patients who present with a tick bite can be treated with a single prophylactic dose of doxycycline (200 mg) to reduce the risk of major adverse outcomes that are commonly seen with the disease, including fever and progression to arthritis and carditis [10]. For early stages of the disease with less severe symptoms including skin lesions or nerve involvement presenting as facial palsy, the recommended treatment is 10-14 days of oral antibiotics for EM and 21-28 days for mild carditis or facial nerve involvement [3]. If the disease progresses to later stages with symptomatic involvement of the joints, a longer course of 28 days of oral antibiotics is used. With meningitis or advanced heart block, a more aggressive approach is indicated with intravenous antibiotics, most commonly IV ceftriaxone [3]. After completion of antibiotic treatment, some patients may still experience persisting symptoms, which should be treated with supportive care to improve their quality of life.

## Conclusions

A moderate suspicion of LD should be maintained in a patient with a tick bite regardless of the appearance of the rash. This is because a characteristic bullseye EM presentation is not seen in most patients presenting with the disease. In medical education, doctors are trained to recognize the classic bullseye rash associated with LD. However, more education is needed to raise awareness among healthcare providers of the different presentations, especially in areas with high rates of LD such as West Virginia. This change can enable healthcare professionals to diagnose and treat this complex condition more quickly, ultimately improving patient outcomes.
